# Supplementary figures and images for: Adapting machine-learning algorithms to design gene circuits
Source: BMC Bioinformatics. 2019 Apr 27;20:214. doi: 10.1186/s12859-019-2788-3 (PMC6487017; doi:10.1186/s12859-019-2788-3)

**A:**

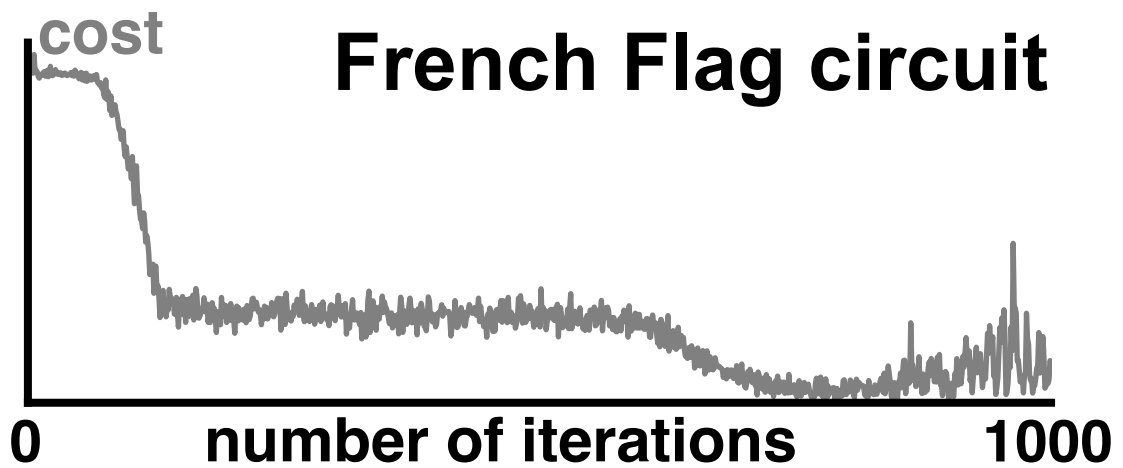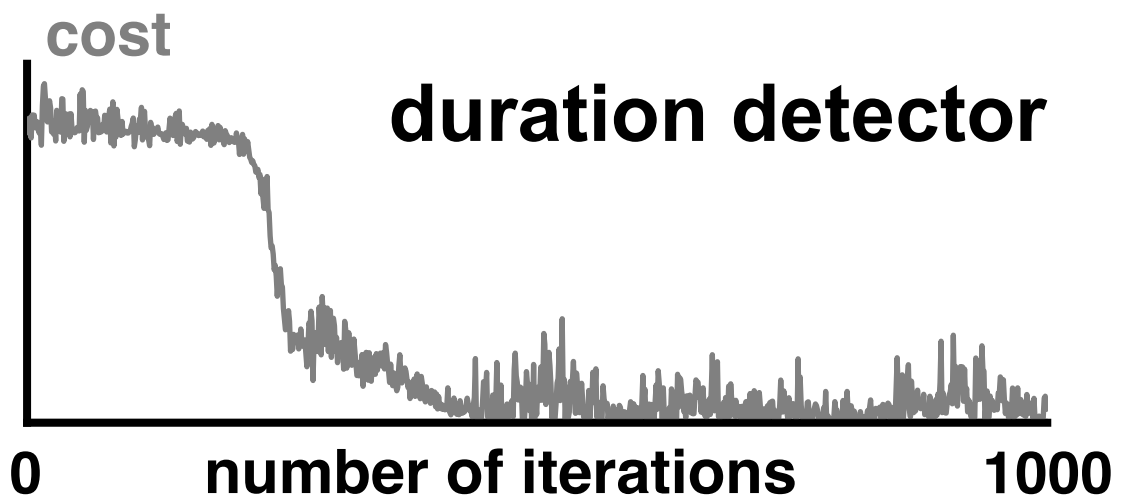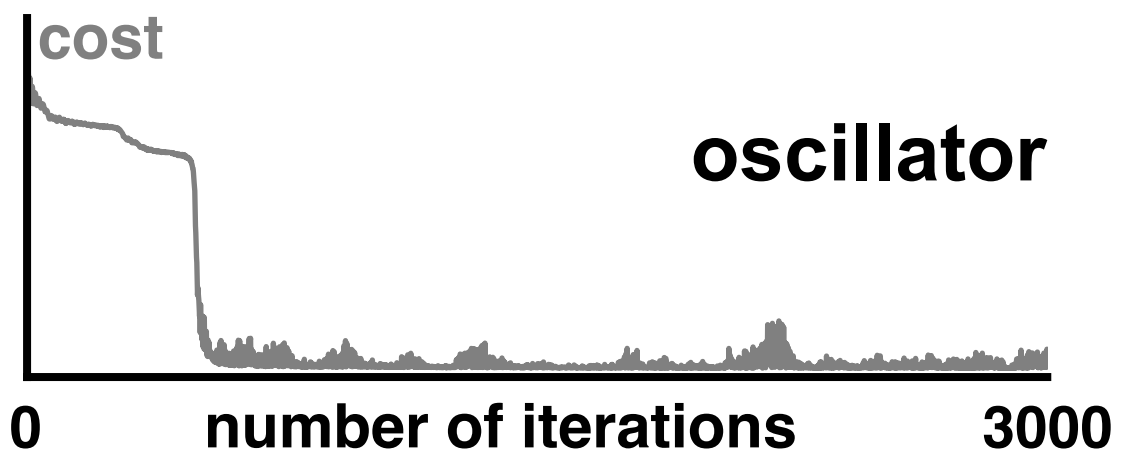

Supplement: Supplementary file 1 — Figure S1. Cost minimization. Example traces of the cost minimization during the optimization procedure. Note, in all cases (and particularly in the oscillator), there are sharp drop-offs in cost, which are likely reflecting bifurcation points in the dynamics. (PDF 177 kb) [file 12859_2019_2788_MOESM1_ESM.pdf]
