# Supplementary material for: Adapting machine-learning algorithms to design gene circuits
Source: BMC Bioinformatics. 2019 Apr 27;20:214. doi: 10.1186/s12859-019-2788-3 (PMC6487017; doi:10.1186/s12859-019-2788-3)
Supplement: Supplementary file 2 — Table S1. Parameter values for networks learned in the main text. Table S2. Algorithm implementation parameters. Table S3. Speed tests. (DOCX 70 kb) [file 12859_2019_2788_MOESM2_ESM.docx]

**Table S1: Parameter values for networks learned in the main text.**

*** denotes these parameters are not learned**

| **Network** | **Input** | **Desired output** | **Learned** $\boldsymbol{W}_{\boldsymbol{ij}}$ | **Learned** $\boldsymbol{k}_{\boldsymbol{i}}$ |
| --- | --- | --- | --- | --- |
| **Switch**  **(Fig 1)** | $x\left( t \right)=A$  $A\sim U(0,2)$  (U is the uniform random distribution) | $\hat{y}=1 \mathrm{for} x>1$  $\hat{y}=0 \mathrm{for} x<1$ | $\left( \begin{matrix} 0 & 0 & 0 \\ -5.5 & 12.9 & 0 \\ 0 & -5.6 & 0 \end{matrix} \right)$ | $\left( \begin{matrix} 1 \\ 1 \\ 1 \end{matrix} \right)^{\boldsymbol{*}}$ |
| **French-flag (Fig 3A)** | $x\left( t \right)=A$  $A\sim U(0,2)$ | $\hat{y}=1 \mathrm{for} 0.5<x<1$  $\hat{y}=0 \mathrm{otherwise}$ | $\left( \begin{matrix} 0 & 0 & 0 \\ -6.3 & 10.8 & 0 \\ -2.2 & -5.8 & 10.0 \end{matrix} \right)$ | $\left( \begin{matrix} 1 \\ 1 \\ 1 \end{matrix} \right)^{\boldsymbol{*}}$ |
| **Duration**  **(Fig 3B)** | $x\left( t \right)=1 \mathrm{for}t=\left[ \frac{1}{6},\frac{1}{6} +\Delta T \right]$  $x\left( t \right)=0 \mathrm{otherwise}$  $\Delta T\sim U(0,\frac{1}{3})$ | $\hat{y}=1 \mathrm{for} \Delta T>\frac{1}{6}$  $\hat{y}=0 \mathrm{otherwise}$ | $\left( \begin{matrix} 0 & -6.1 & 0 \\ -7.8 & 6.1 & 0 \\ 0 & -5.0 & 0 \end{matrix} \right)$ | $\left( \begin{matrix} 1 \\ 1 \\ 1 \end{matrix} \right)^{\boldsymbol{*}}$ |
| **Oscillator (Fig 3C)** | $x\left( t \right)= 0$ | $\hat{y}\left( t \right)=1+cos(\omega t)$ | $\left( \begin{matrix} 3.6 & -3.6 & 0 \\ 0 & 3.2 & -3.7 \\ -6.3 & 0 & 5.1 \end{matrix} \right)$ | $\left( \begin{matrix} 3.3 \\ 7.3 \\ 1.0 \end{matrix} \right)$ |
| **Counter**  **(Fig 5)** | $N$ pulses,  Amplitude: $A\sim U(1,2)$,  Duration: $\Delta T\sim U\left( 10\delta t, 30\delta t \right)$  Separation: $\delta T\sim exp(1/\lambda)$  Pulse rate: $\lambda\sim U(0,100\delta t)$ | $\int_{0}^{T} \hat{y}\left( t \right)dt=N$ | $\left( \begin{matrix} 0 & -4.0 & 0 \\ -7.2 & 3.2 & 4.1 \\ -2.5 & -10.2 & 6.2 \end{matrix} \right)$ | $\left( \begin{matrix} 17.6 \\ 27.3 \\ 28.5 \end{matrix} \right)$ |

**Table S2: Algorithm implementation parameters.**

N: number of iterations

B: batch size

$\lambda$: regularization parameter

$\epsilon$: pruning parameter

Note that in some cases we perform multiple optimization procedures, starting with a small batch size to increase noise in the early steps. From our experimentation, the iteration number, N, and batch size, B, can be varied significantly and the algorithm still works – one simply needs to increase *N* and *B* so that enough training samples are used. Here we report the parameters we used to generate the figures in the main text, but expect the precise values to be unimportant, and should be chosen/modified by the user.

| **Network** | **Training** | **Regularization** | **Pruning** |
| --- | --- | --- | --- |
| **Switch**  **(Fig 1)** | $N=400$  $B=128$  $\lambda=0.0$ | $N=1000$  $B=128$  $\lambda=0.56$ | $N=1000$  $B=128$  $\lambda=0.0$  $\epsilon=1$ |
| **French-flag (Fig 3A)** | $N=1000$, $B=2$,  $N=1000$, $B=128$,  $\lambda=0.0$ | $N=1000$  $B=2$  $\lambda=0.02$ | $N=1000$  $B=64$  $\lambda=0.0$  $\epsilon=1$ |
| **Duration**  **(Fig 3B)** | $N=1000$  $B=64$  $\lambda=0.0$ | $N=1000$  $B=2$  $\lambda=0.02$ | $N=1000$  $B=64$  $\lambda=0.0$  $\epsilon=1$ |
| **Oscillator (Fig 3C)** | $N=1000$  $B=1$  $\lambda=0.0$ | $N=400$  $B=1$  $\lambda=0.02$ | $N=1000$  $B=1$  $\lambda=0.0$  $\epsilon=1$ |
| **Counter**  **(Fig 5)** | $N=10000$, $B=2$,  $N=3000$, $B=128$,  $N=10000$, $B=64$,  $\lambda=0.0$ | $N=1000$  $B=64$  $\lambda=0.78$ | $N=1000$  $B=64$  $\lambda=0.0$  $\epsilon=1$ |

**Table S3: Speed tests.**

Compute time on Macbook air, 1.3GHz Intel Core i5 CPU, with 8GB 1600 MHz DDR3 RAM for different software implementations. Given is the time to perform 1000 iterations of the learning algorithm, with a batch size of 64, simulating 300 timesteps. We expect speed to be significantly improved using GPUs.

| **Implementation** | **Compute time (s)** |
| --- | --- |
| **Theano**  **(unoptimized)** | 48.5 |
| **Theano**  **(compiled for optimization)** | 28.5 |
| **Tensorflow** | 309.0 |
